# Poly(di-allyl phthalate) hybrid nanocomposites with magnetic conductive coupling for absorption dominated EMI shielding

**DOI:** 10.1038/s41598-025-33772-3

**Published:** 2026-01-19

**Authors:** Ahmed Saeed Abo Elfath, Hamada Abd Elwahab, Medhat E. Owda, Ahmed Shalaby, Hussein Oraby

**Affiliations:** 1 Chemistry Department, Faculty of Science (boys), Al-Azhar University, Cairo, Egypt; 2 Science and Technology Centre of Excellence, Cairo, Egypt; 3 University of Chemical Technology and Metallurgy, 8 Kl. Ohridski blvd, 1756 Sofia, Bulgaria; 4 Department of Chemical Engineering, Military Technical College, Cairo, Egypt

**Keywords:** Electromagnetic interference, Shielding, Poly di-allyl phthalate matrix, Fe_3_O_4_@RGO nanocomposite, Materials science, Nanoscience and technology, Physics

## Abstract

Electromagnetic interference (EMI) remains a critical challenge for modern electronic systems, driving the need for lightweight materials with efficient shielding capabilities. In this work, Fe_3_O_4_@RGO hybrid nano-fillers were synthesized via a co-precipitation route and incorporated into a poly(di-allyl phthalate) (PDAP) matrix to produce composite films with tunable dielectric–magnetic coupling. Structural analyses confirmed the uniform decoration of Fe_3_O_4_ nanoparticles on RGO sheets and their homogeneous dispersion within the PDAP network. The calculated electromagnetic parameters (ε′ ≈ 12, ε″ ≈ 4, µ′ ≈ 1.3, µ″ ≈ 0.3 at 10 GHz) reveal strong dielectric and magnetic loss channels that support an absorption-dominated shielding mechanism. The optimal composite (PMR20) achieved a total shielding effectiveness of ~ 31.5 dB across the X-band, corresponding to > 99.9% attenuation of incident radiation, with SEA contributing the major portion of the overall SE. The synergy between conductive RGO pathways, magnetic relaxation of Fe_3_O_4_, and interfacial polarization within PDAP underpins the enhanced attenuation behavior. This study demonstrates PDAP as a promising and underexplored thermosetting host for hybrid EMI shielding materials, offering a platform for lightweight and high-performance electromagnetic protection.

## Introduction

Due to rapid advancements in digital technologies and electronic network infrastructure in recent years, smart urban architecture is rapidly developing, relying heavily on sensing technology and wireless communication networks^1^. As a result of these developments, however, a significant problem concerning electromagnetic interference (EMI) has emerged and is growing in terms of the extent of its influence on the operation of electronic equipment^2^, a form of electromagnetic pollution that will impair the functioning of many electronic devices and reduce their operating efficiencies^3^ unless a mitigation strategy such as the use of shielding materials is employed^3^. Beyond reducing the effects of EMI, sophisticated materials engineering also enhances the reliability and life expectancy of all electronic architectures^4^. Metallic alloys and compounds have traditionally represented the most common choice of materials used for EMI shielding^5^; unfortunately, they represent a limited class of materials because of their high mass density, susceptibility to corroding processes, and costly manufacturing process^5^. The properties of carbon based materials present an attractive alternative with respect to these limitations as they possess a high level of resistance to corrosion, low mass density, high levels of electrical conductivity, and excellent thermal stability^6^. Therefore, due to their favorable properties related to cost, performance, and efficiency in microwave absorption, these materials can be utilized to design lightweight shielding structures^7^. There are two primary mechanisms that provide EMI shielding: (i) reflection which relies upon electrical conductivity to absorb the electric field components of electromagnetic waves and attenuate them and (ii) absorption which is provided by magnetic materials that convert the magnetic field components of electromagnetic waves into heat^8^. While conventional shielding relies on heavy metal matrices, polymer-based nano-composites have emerged as attractive candidates because of their low weight, flexibility, corrosion resistance, and ease of fabrication^9^. Of all the other electrically-conductive fillers that have been studied such as carbon black, carbon nanotubes, carbon fibers, graphite, graphene, and reduced graphene oxide (RGO)^10^ graphene has attracted the most attention because of its unique combination of physical properties including electrical, mechanical, and thermal properties^11,12^; and because it is considered to be one of the thinnest, strongest nanomaterials available^13^. The SE of graphene-based materials can be enhanced by modifying their electromagnetic properties through adjusting both dielectric and magnetic properties of these materials. The adjustment of dielectric and magnetic properties are typically achieved by adding functionalized nanoparticles onto the surface of graphene sheets, such as Fe_3_O_4_ or BaTiO_3_, in order to modify permittivity and permeability^14,15^. Several studies have reported graphene-enhanced polymer composites exhibiting strong absorption-dominated shielding performance. For example, Wang et al. demonstrated that graphene/epoxy composites could achieve > 30 dB shielding in the X-band through enhanced dipole polarization and percolated conductive pathways^16^. More recently, hybrid graphene systems combining dielectric and magnetic components have shown superior performance due to synergistic loss mechanisms. Katheria et al. integrated Fe_3_O_4_ nanoparticles onto graphene sheets to produce a magnetic–conductive hybrid that reached 35–40 dB in the X-band^17^. Comparable results were obtained by Wei et al. using Fe_3_O_4_@rGO within polyurethane, achieving enhanced SEA values driven by magnetic relaxation^18^. Magnetic fillers used in EMI shielding include Ni nanowires, permalloy alloys, ferrites, and spinel oxides^19^, each offering distinct magnetic loss mechanisms such as natural resonance and eddy-current damping. Fe_3_O_4_-based hybrids are particularly attractive due to their high stability, low cost, and strong magnetic relaxation, making Fe_3_O_4_@RGO a competitive class of magnetic–conductive fillers^20^. Fe_3_O_4_ nanoparticles are considered to be among the best candidates for use due to their strong spin-polarization at room temperature, suitable magnetic behavior, low toxicity, and good chemical compatibility with polymer matrices^21,22^. Furthermore, Fe_3_O_4_ nanoparticles show a skin depth effect and moderate electrical conductivity which ensures the good absorption of the electromagnetic radiation^22^. The synergistic effect of the RGO together with the Fe_3_O_4_ enhances this electromagnetic interference (EMI) shielding due to the effect of the electrical conductivity of the RGO phase and the magnetic loss due to the Fe_3_O_4_ phase^23^. For effective electromagnetic attenuation it is essential that the polymer composites have sufficient electrical conductivity and permeability along with proper structure retention which can be achieved by the addition of conducting and magnetic fillers to the polymer host^24^. Increase in filler concentration leads to a corresponding increase in EMI shielding until the (optimum) concentration is obtained when any further loading will tend to lessen the structural integrity of the composite^25^. Recent studies have demonstrated the potential of Fe_3_O_4_@RGO hybrid nanostructures for EMI shielding due to their combined conductive and magnetic loss characteristics. Fe_3_O_4_ nanoparticles introduce magnetic dipole resonance and domain-wall relaxation, while RGO provides conductive pathways for dielectric and interfacial polarization losses^17^. Several reports have shown Fe_3_O_4_@RGO-based polymer composites achieving moderate-to-high shielding performance in the X-band, confirming the relevance of this hybrid design^26^. However, most studies have focused on flexible matrices such as epoxy, silicone, or polyethylene-based systems, while thermosetting polyesters such as PDAP remain largely unexplored^27^. These gaps highlight the need to investigate Fe_3_O_4_@RGO hybrids within new polymer hosts and to optimize filler ratios to achieve synergistic absorption-dominated EMI shielding. Some of the Fe_3_O_4_@RGO/polymer composites have been made and the literature shows that most of the work has been done on their applications in epoxies and polyurethanes which are known to have poor conductivity and interface adhesion. In contrast the poly (di-allyl phthalate) (PDAP) polymer has a rigidity due to cross-linked ester structure and has been shown to have the ability to improve the interfacial polarizability and dielectric enhancement. However, its potential as an EMI shielding matrix remains largely unexplored. PDAP was selected as the polymer host due to its high thermal stability, cross-linked structure, and strong dielectric strength, which are advantageous for EMI shielding materials. The ester and aromatic groups in PDAP exhibit good compatibility with Fe_3_O_4_@RGO hybrids through hydrogen bonding and dipole–dipole interactions with residual hydroxyl, epoxy, and carbonyl functionalities on RGO and Fe_3_O_4_ surfaces. Such interfacial affinity enhances nano-filler dispersion and promotes interfacial polarization, which contributes to improved dielectric losses and absorption-dominated shielding. Therefore, the present study aims to synthesize and characterize Fe_3_O_4_@RGO/PDAP composites and evaluate their electromagnetic performance within the X-band frequency range (8–12 GHz). The novelty of this work lies in employing PDAP as a new polymer host to exploit its cross-linked ester structure for improved interfacial interactions with Fe_3_O_4_@RGO hybrids. The study demonstrates how the magnetic–dielectric coupling between Fe_3_O_4_ and RGO within PDAP leads to efficient absorption-dominated EMI shielding behavior, offering a new material platform for lightweight and high-performance EMI protection.

## Theory of EMI shielding

**Fig. 1:**
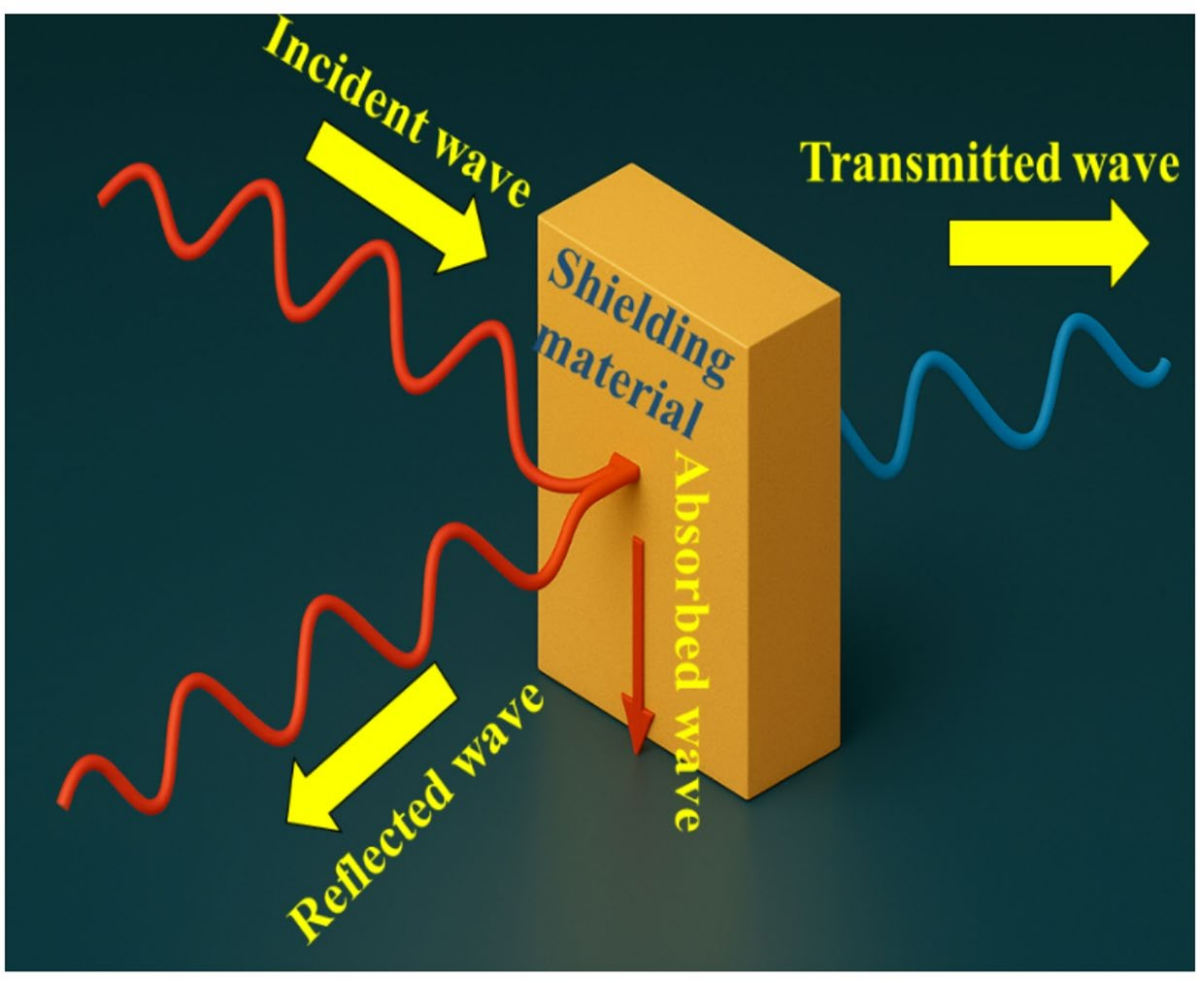
Mechanism of shielding efficiency.

The ratio of the electric or magnetic field intensity before and after transmission through a shielding material defines the shielding effectiveness (SE), which can be expressed as shown in Eq. 1^28^.

1 $${\mathrm{SE}} = - 20{\mathrm{log}}[{\mathrm{E}}_{{\mathrm{T}}} /{\mathrm{E}}_{{\mathrm{i}}} ] = - 20{\mathrm{log}}[{\mathrm{H}}_{{\mathrm{T}}} /{\mathrm{H}}_{{\mathrm{i}}} ] = - 10{\mathrm{log}}[{\mathrm{P}}_{{\mathrm{T}}} /{\mathrm{P}}_{{\mathrm{i}}} ]$$

where *P*, *E*, and *H* represent the electromagnetic wave power, electric field, and magnetic field, respectively. Subscripts *T* and *i* denote transmitted and incident waves. Shielding effectiveness is dependent on many different variables; primarily the amount of physical separation between the source of the interference and the shielded area, the thickness of the shield, the frequency being shielded against, and the electric and magnetic characteristics of the material used to construct the shield. Shielding effectiveness is generally quantified in terms of Decibel levels (dB). Variables that affect the magnitude of EMI reduction include wavelength, the proximity of the interference source to the shielded area, the physical dimensions and inherent properties of the shielding material. There are three basic mechanisms that govern how electromagnetic waves interact with shielding materials. As indicated in Fig. 1, when an electromagnetic wave enters into a shielding material some of it will be reflected off the front surface, a portion of it will be absorbed by the material itself, and a portion of it may exit the material and reflect off the rear surface. In accordance with this behavior, the total shielding effectiveness (SET) of the material can be calculated based upon a combination of the reflection loss (SER), the absorption loss (SEA), and a correction factor (M) which represents the number of times that the remaining portions of the electromagnetic wave internally reflects. This relationship is outlined in Eq. 2:

2 $${\mathrm{SET}} = {\mathrm{SEA}} + {\mathrm{SER}} + {\mathrm{M}}$$

When absorption loss exceeds a value of 10 dB, then the contribution of the multiple reflection component (M), is reduced to negligible amounts and can be simple computational models for plane waves, current induced in conducting media are lost as heat rather than contributing to additional reflections^29^. Reflections occur due to impedance discontinuities at the interface of the incoming wave and the shield surface. As SEA approaches values of approximately 6 dB, and beyond, reflection correction factors become irrelevant especially at low frequencies (less than 20 kHz)^30^. Alternatively, shielding characteristics can be evaluated using two-port Vector Network Analyzer methods, where the reflection and transmission characteristics of the electromagnetic wave are determined by S-parameters. When a wave is propagated from Port 1 to Port 2, it interacts with the shield through reflection, absorption and transmission mechanisms; therefore, the S_11_ and S_21_ represent the reflected and transmitted portions of the incident wave, respectively^31^. Consequently, the relationships between transmittance (T), reflectance (R), and absorbance (A) and their corresponding S-parameters are expressed through Eqs. (3–5)^32^:

3 $${\mathrm{T}} = \left[ {{\mathrm{E}}_{{\mathrm{T}}} /{\mathrm{E}}_{{\mathrm{i}}} } \right]^{{\mathrm{2}}} = {\mathrm{S}}_{{{\mathrm{12}}}} ^{{\mathrm{2}}} ~ = {\mathrm{S}}_{{{\mathrm{21}}}} ^{{\mathrm{2}}}$$

4 $${\mathrm{R}} = \left[ {{\mathrm{E}}_{{\mathrm{R}}} /{\mathrm{E}}_{{\mathrm{i}}} } \right]^{{\mathrm{2}}} = {\mathrm{S}}_{{{\mathrm{11}}}} ^{{\mathrm{2}}} ~ = {\mathrm{S}}_{{{\mathrm{22}}}} ^{{\mathrm{2}}}$$

5 $${\mathrm{A}} = {\mathrm{1}}{-}{\mathrm{T}}{-}{\mathrm{R}}$$

Here, *A* represents the absorbed portion of the electromagnetic power. Even when multiple reflections between the front and back interfaces are minimal, the fraction of the effective incident energy entering the shield is determined by (1 – R). Consequently, the effective absorption can be expressed as shown in Eq. 6^33^:

6 $${\mathrm{A}}_{{{\mathrm{eff}}}} = \left( {{\mathrm{1}}{-}{\mathrm{T}}{-}{\mathrm{R}}} \right)/\left( {{\mathrm{1}}{-}{\mathrm{R}}} \right)$$

Using power balance relationships, the reflection (*SER*), absorption (*SEA*), and total shielding (*SET*) effectiveness can be calculated in terms of *R*, *T*, and *A* as shown in Eqs. (7–9):

7 $${\mathrm{SE}}_{{\mathrm{R}}} = {\mathrm{1}}0{\mathrm{log}}\left( {{\mathrm{1}}{-}{\mathrm{R}}} \right)$$

8 $${\mathrm{SE}}_{{\mathrm{A}}} = {\mathrm{1}}0{\mathrm{log}}\left( {{\mathrm{1}}{-}{\mathrm{A}}} \right) = {\mathrm{1}}0{\mathrm{log}}\left[ {{\mathrm{T}}/\left( {{\mathrm{1}}{-}{\mathrm{R}}} \right)} \right]$$

9 $${\mathrm{SE}}_{{\mathrm{T}}} = {\mathrm{1}}0{\mathrm{log}}\left( {{\mathrm{1}}{-}{\mathrm{R}}} \right) + {\mathrm{1}}0{\mathrm{log}}\left[ {{\mathrm{T}}/\left( {{\mathrm{1}}{-}{\mathrm{R}}} \right)} \right]$$

## Experimental work

### Materials

PDAP (Average M.W. 65000 (GPC), purity 69%), Graphite powder (G) (99.5%), Ammonium iron (II) sulfate hexahydrate (NH_4_)_2_Fe(SO_4_)_2_·6H_2_O (Mw − 392.14 g/mol, purity 98.5%), Iron (III) chloride FeCl_3_.6H_2_O (Mw − 270.30 g/mol, purity 98.5%), sodium hydroxide (NaOH, 99.5%), Potassium permanganate (KMnO4, 98.5%), Hydrogen peroxide (H_2_O_2_, 30%), Sodium Borohydride ( NaBH_4_, 99.5%), Hydrochloric acid (HCl, 36%) and Sulfuric acid (H_2_SO_4_, 98%) were obtained from Alpha Chemicals, Haryana; India. All chemicals were used as purchased without further treatment or purification.

### Preparation of reduced graphene oxide (RGO)

The preparation of graphene oxide (GO) involved oxidizing graphite powder via a modified Hummers’ method^34^. During the process, the graphite powder was first mixed into concentrated sulfuric acid (H_2_SO_4_) with ongoing agitation, after which potassium permanganate (KMnO_4_) was introduced gradually while ensuring the mixture stayed below 20 °C to avoid excessive heat and over-oxidation. Deionized water was then added step by step to dilute the reaction, followed by the careful incorporation of hydrogen peroxide (H_2_O_2_, 30%) to halt the oxidation, which produced a vivid yellow hue indicative of GO formation. The suspension was subjected to several washes with distilled water and ethanol, as well as 5% hydrochloric acid until the pH of the solution was neutral. The residue was filtered off and dried at 60 °C giving the pure GO. The exfoliation of dried graphite oxide was obtained by ultra-sonication for three hours giving a uniform suspension of GO dispersions^35^. A chemical reduction of this suspension of GO was then carried out with sodium borohydride or NaBH_4_ resulting in the synthesis of reduced graphene oxide (RGO) which has excellent electrochemical properties with a regenerated framework of π-conjugated units^36^.

### Synthesis of Fe_3_O_4_ and Fe_3_O_4_@RGO hybrid nanocomposites

The preparation of Fe_3_O_4_ and Fe_3_O_4_@RGO nanocomposites was completed using a chemical co-precipitation method^37^ for both processes. The general process involved a solution containing a combination of ferric chloride hexahydrate (FeCl_3_·6 H_2_O) and ammonium iron (II) sulfate hexahydrate ((NH_4_)_2_Fe(SO_4_)_2_⋅6H_2_O), which was created by dissolving these two compounds in 25 mL of DI water with a molar ratio of 2:1. This solution was then stirred magnetically for several hours until the temperature reached 120 °C and then slowly added to 1 L of 1.5 M NaOH solution that contained a variable amount of RGO (0, 10, 20, 30, and 40 wt%). At the time of mixing of the solutions, there was an immediate formation of a black precipitate indicating successful co-precipitation of Fe_3_O_4_ nanoparticles onto RGO sheets. After completion of the reaction, all of the products were separated from solution by use of a magnetic separator; washed 3 times each with ethanol and DI water; and dried under vacuum at 60 °C overnight. These resulting nanocomposites received designations of Fe_3_O_4_, MR10, MR20, MR30, and MR40, aligned with RGO contents of 0%, 10%, 20%, 30%, and 40%, respectively.

### Synthesis of Fe_3_O_4_@RGO/PDAP composites

The Fe_3_O_4_@RGO/PDAP composites were fabricated through a solvent-assisted blending method to ensure homogeneous distribution of the hybrid filler and to avoid premature crosslinking of the PDAP resin. A predetermined amount of Fe_3_O_4_@RGO powder was dispersed in 40 mL acetone using probe sonication (576 W, 25 min). The PDAP resin was added gradually under magnetic stirring and mixed for 1 h to ensure complete wetting of the filler. The solvent was then removed under reduced pressure, and the resulting viscous mixture was poured into Teflon molds and cured at room temperature for 24 h. Four composite samples were prepared with different hybrid filler loadings and are denoted as PMR10, PMR20, PMR30, and PMR40 corresponding to 10, 20, 30, and 40 wt% Fe_3_O_4_@RGO, respectively. It is important to clarify that PDAP is a thermosetting resin and cannot be processed by melt blending due to the risk of in-situ thermal crosslinking during heating. For this reason, no melt-processing was used in this study. All mixing steps were performed at room temperature using a solvent-assisted blending method, ensuring that PDAP remained un-crosslinked during filler incorporation. Crosslinking occurred only during the final curing stage inside the mold, thereby avoiding any premature polymer network formation during composite preparation.

### Comprehensive structural, morphological, DC electrical conductivity and electromagnetic characterization

The physical and electromagnetic properties of the developed composites were evaluated through the use of several analysis techniques to characterize their structural, morphological, magnetic, and electromagnetic characteristics. The images obtained from Transmission Electron Microscopy (TEM) of the developed composites were taken on a Thermo Scientific Talos F200i system (USA) that produced images in a voltage range of 20 to 200 kV and resolutions of less than or equal to 0.10 nm. The samples for TEM characterization were prepared by suspending a diluted composite dispersion onto a carbon coated ultrathin copper grid, followed by air-drying the samples at room temperature. Patterns of X-Ray Diffraction (XRD) were collected using a PANalytical X’Pert Pro Diffractometer (UK) that used both Cu Kα radiation (λ = 1.54Å) and Mo Kα radiation (λ = 0.71Å). Raman Spectroscopy measured the wavenumbers of the 400–3000 cm^−1^ region at a resolution of 4 cm^−1^, using a Bruker SENTERRA II spectrometer (USA) equipped with a 785 nm laser at 10mW. FT/IR Spectra were collected to determine the chemical bonding between the PDAP matrix and the Fe_3_O_4_@RGO fillers using a Nicolet iS50 instrument (Thermo Scientific, USA) that provided a resolution of 0.09 cm^−1^ over the 15–27,000 cm^−1^ range. Magnetic attributes such as Saturation Magnetization (Ms), Magnetic Remanence (Mr), and Coercivity (Hc) for the Fe_3_O_4_, MR10, MR20, MR30, and MR40 specimens were determined by Vibrating Sample Magnetometry (VSM, Model UP200S, Hielscher, Germany). Surface morphology and the distribution of the filler material within the polymer matrix were inspected by Scanning Electron Microscopy (SEM, ZEISS EVO 15, Carl Zeiss, Berlin, Germany). The DC electrical conductivity (σ) of the Fe_3_O_4_@RGO/PDAP composites was measured using a standard four-point probe station (Keithley 2400 SourceMeter coupled with a Jandel RM3000 Four-Point Probe System). The measurements were performed in accordance with ASTM F76-08 (Standard Test Method for Measuring Resistivity of Thin Conductive Materials). Rectangular specimens (typically 20 × 10 × 3–5 mm^3^) were cut from the cured composite sheets. A constant current was applied through the outer probes while the voltage drop between the inner probes was recorded. Each measurement was repeated three times at different surface positions to ensure reproducibility, and the average DC conductivity was calculated according to the standard geometric correction factors specified in ASTM F76-08. The DC conductivity values (σ) were obtained for all composite formulations: PMR10, PMR20, PMR30, and PMR40. These values were later correlated with the EMI shielding performance and with the calculated dielectric parameters in the Results and Discussion section. The electromagnetic interference (EMI) shielding capability of the developed composites was assessed in the X-band frequency range (8–12 GHz) using a vector network analyzer (N9918A Field Fox, Agilent Scientific, USA). Rectangular-shaped samples measuring 22.86 × 10.16 mm^2^ were prepared to fit the WR-90 waveguide setup. The recorded scattering parameters included S11, S21, S22, and S12, from which the total shielding effectiveness (SET), reflection loss (SER), and absorption loss (SEA) were calculated. Three sets of measurements were performed for each specimen for consistency purposes, the results shown represent the average value of the three measurements; the uncertainty of the measured quantities of the EMI shielding efficiency and electrical conductivity is < ± 3%. A full NRW retrieval from S-parameters was not available with the current instrumentation; therefore, an analytical NRW-based approach was employed, where the complex permittivity and permeability were calculated from EMI shielding data using classical Maxwell relations.

## Results and discussion

### Filler characterization

#### X-ray diffraction analysis

**Fig. 2:**
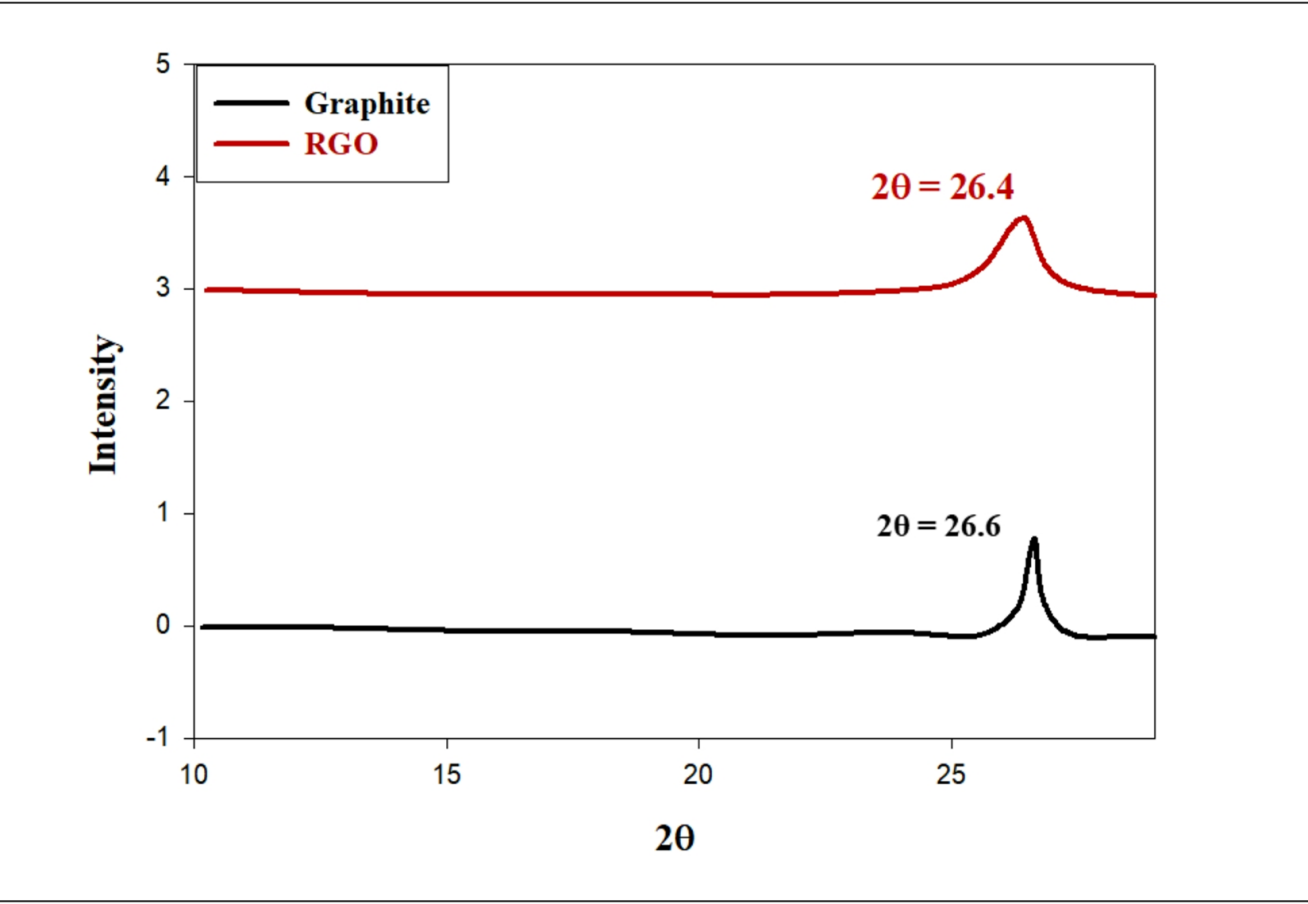
XRD patterns of graphite and reduced graphene oxide.

The XRD patterns in Fig. 2 show that both RGO and as-received graphite produced strong peaks associated with their respective d-spacings. Graphite gave rise to a large signal at 26.6° (002) showing a d-spacing of approximately 3.5 Å. Following the oxidation process, a very weak peak was observed at 27.32° (d = 3.57 Å) demonstrating expanded d-spacing due to the inclusion of H_2_O molecules between adjacent layers and the introduction of O-functional groups on the graphite layers^38^.

The interlayer spacing (*d*) was calculated using Bragg’s law, as indicated in Eq. (10):

10 $${\mathrm{n}}\lambda = {\text{2d sin}}\theta$$

**Fig. 3:**
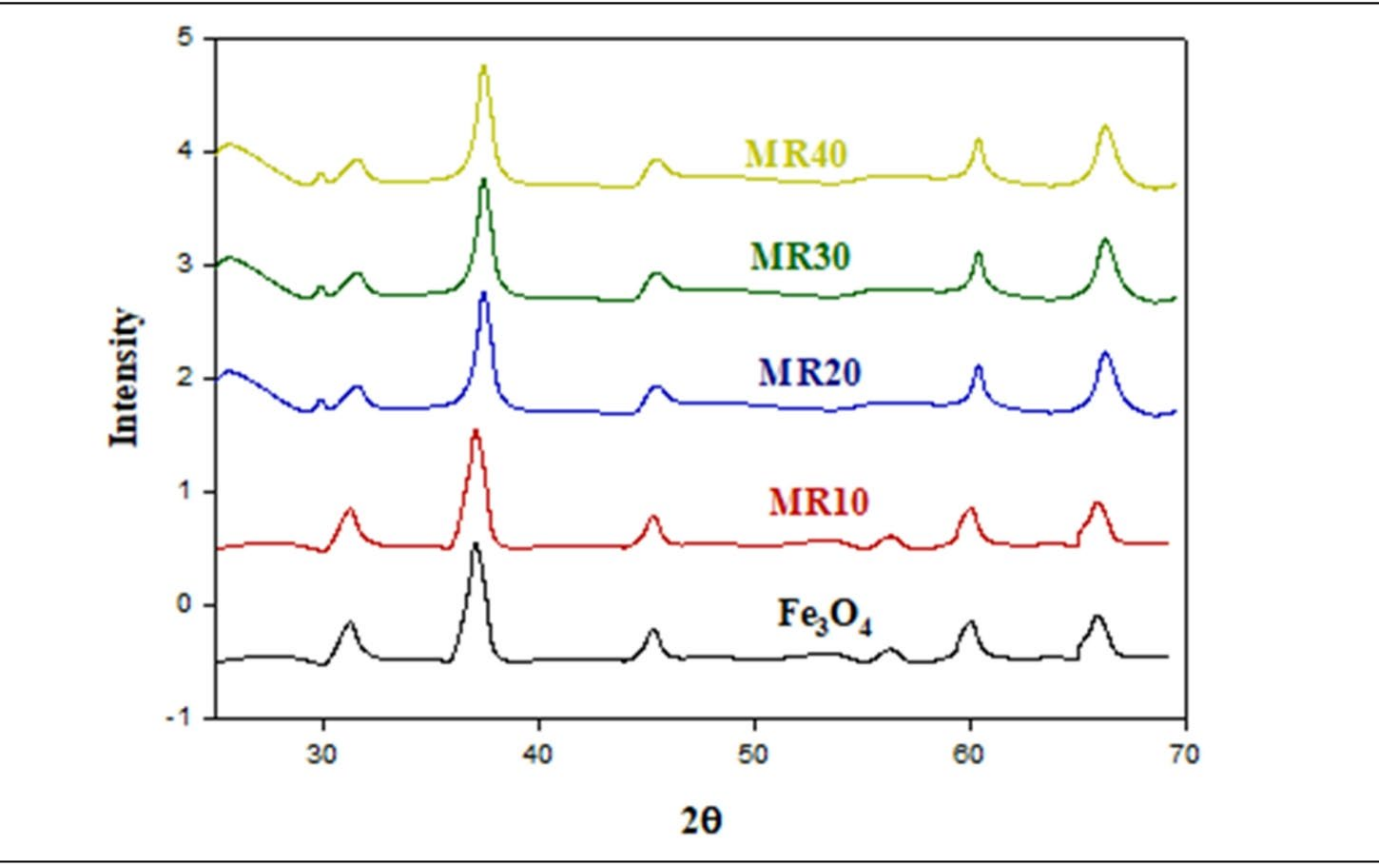
The XRD spectra for Fe_3_O_4_, MR10, MR20, MR30, and MR40.

Where λ is the wavelength of the X-rays; θ is the angle of diffraction; d is the spacing between crystallographic planes^39^. Using Bragg’s Law, we determined the d-spacing for unmodified graphite to be 3.41Å at 2θ = 26.4°. We also determined that a large decrease in intensity of the (002) peak at 2θ = 27.32° resulted from the chemical reduction of graphene oxide using sodium borohydride, which indicated the partial restoration of the conjugated graphitic structure. The d-spacings were equivalent for both graphite and reduced GO, indicating that the majority of oxygen containing functional groups were removed from the RGO via the modification of the Hummers oxidation process^40^. In Fig. 3, we have provided the XRD patterns for pristine Fe_3_O_4_ nanoparticles, RGO and the Fe_3_O_4_@RGO nanocomposites (MR10, MR20, MR30, and MR40). There is a broad diffraction pattern located near 2θ = 25° that corresponds to the (002) plane of RGO, and there is a strong increase in intensity of this peak as the amount of RGO increases to reach an extreme in the MR40 sample. Conversely, the pure Fe_3_O_4_ has sharp diffraction peaks that indicate no shift in the lattice parameters relative to the original spinel structure^41^. Notable peaks emerged at 2θ values of 31°, 37°, 43°, 57°, and 65°, linked to the (220), (311), (400), (511), and (440) planes of Fe_3_O_4_, respectively. These characteristics indicate that the material is an inverse spinel magnetite (JCPDS card number 19–0629). The peaks’ loss of clarity and strength as the amount of RGO increased suggested that there were reduced crystalline features due to strong interactions between RGO layers and structured Fe_3_O_4_ domains^42^, such that a determination of the average crystallite dimension (L) could be made by applying Scherrer’s equation as defined in Eq. 11:

11 $${\mathrm{L}} = {\mathrm{k}}\lambda /\left( {\beta {\text{ cos}}\theta } \right)$$

where L is the crystallite size (nm), *k* is the shape factor (~ 0.9), *λ* is the X-ray wavelength (nm), and *β* is the full width at half maximum (FWHM) of the most intense peak^43^. The observed broadening of Fe_3_O_4_ peaks in the composites further supports the nanoscale crystallite dimensions and the successful hybridization of Fe_3_O_4_ with RGO, yielding well-dispersed magnetic domains within the conductive carbon network.

The calculated crystallite domain sizes of all the samples are shown in Table 1. Pure Fe_3_O_4_ has the largest average crystallite size (29.6 nm) which progressively decreases, due to the increased RGO content, down to ∼5.44 nm for MR40 composite. This considerable reduction in crystallite size at higher RGO loadings suggests the incorporation of RGO limits the growth and aggregation of Fe_3_O_4_ nanoparticles during synthesis. The decrease in crystallinity and crystallite size of the material is indicative of the strong interfacial interactions of Fe_3_O_4_ with the conductive RGO sheets, which serve as effective nucleation and confinement sites for the feeder nanoparticle growth. In effect it is shown that the addition of RGO at higher concentrations promotes dispersion of the smaller particles, resulting in a greater degree of structural homogeneity of the hybrid composite, as indicated by the broadening of the observed XRD peaks.

**Table 1:** The crystalline domain size (L, nm) for the different Fe_3_O_4_/RGO composites.

#	Composition	β (Radians)	L(nm)
1	Fe_3_O_4_	0.0047	29.7
2	MR10	0.0076	20.5
3	MR20	0.0106	13.66
4	MR30	0.0143	8.14
5	MR40	0.0221	5.44

#### Raman spectroscopy analysis

**Fig. 4:**
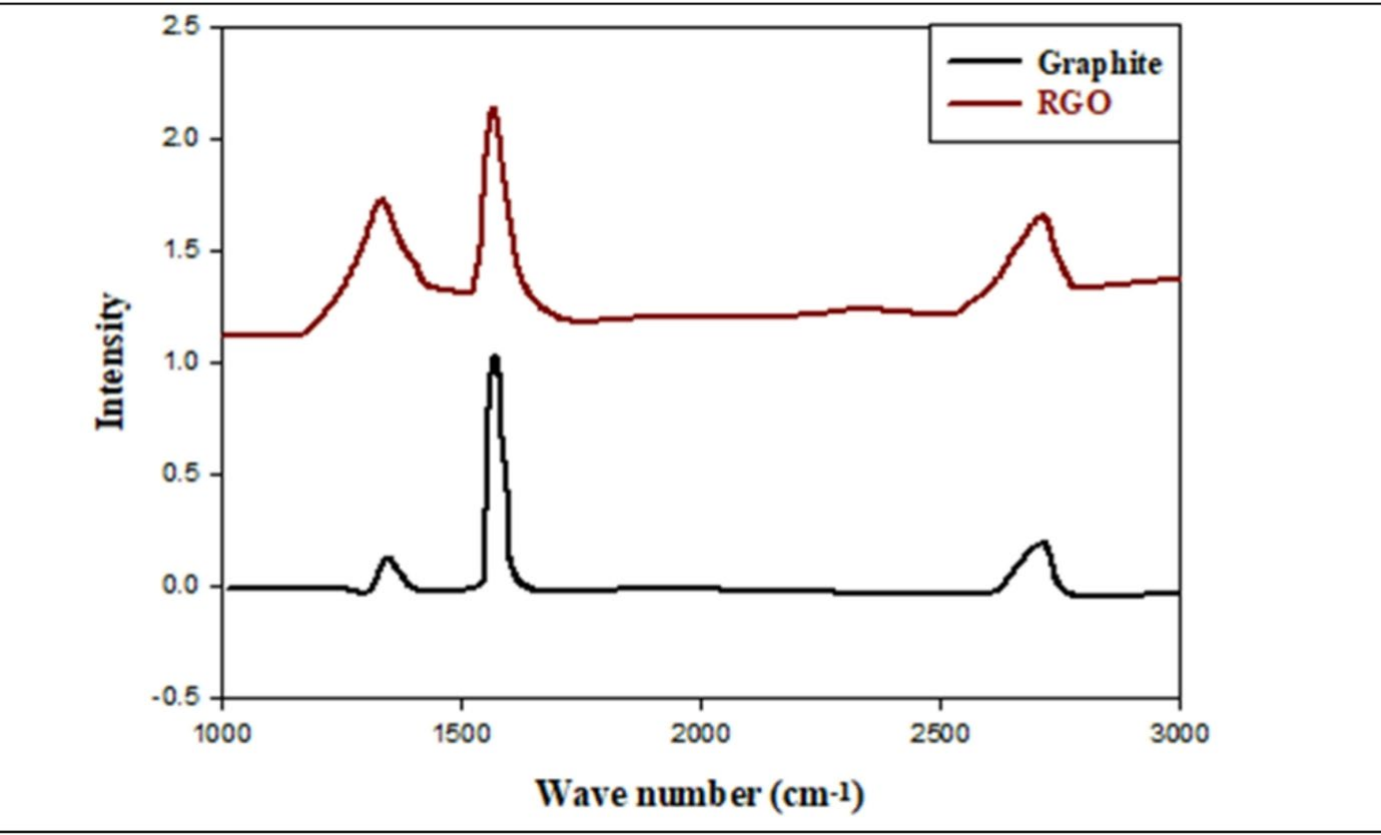
Raman spectra for graphite and RGO.

The Raman Spectra are displayed in Fig. 4 for graphite as well as reduced graphene oxide (RGO) as well as the spectra show the typical 3 bands associated with *sp*^*2*^ hybridized carbon graphitic material; namely the 2D, D, and G bands. The G Band at 1570 cm^−1^ is indicative of the in-plane motion of *sp*^*2*^ hybridized carbon atoms in the hexagonal structure of graphite^44,45^ and represents the universal signature of *sp*^*2*^ carbon systems including graphite, CNTs, fullerenes, and amorphous carbon. Upon chemical reduction, the G Band in RGO shifts slightly to 1574 cm^−1^ and diminishes significantly in intensity as an indication of the incomplete recovery of *sp*^*2*^ domains. The D Band, indicative of breathing motions of *sp*^2^ carbon rings that are activated via lattice disorder or *sp*^*3*^ defect formation, is located at 1339 cm^−1^ in graphite and at 1344 cm^−1^ in RGO^46^, and indicates increased defect and boundary region creation during the oxidation and reduction stages as evidenced by the incomplete removal of oxygen containing functional groups. Graphite exhibits the 2D band at 2723 cm^−1^ while RGO exhibits the same band at 2711 cm^−1^ but with increased symmetry and strength indicating the emergence of a few layered sheets. The I_2_D/IG band intensity ratio provides a qualitative estimate of graphene layer thickness^47^; according to this ratio, the original graphite consists of > 20 layers, in contrast to the estimated 7–9 layers present in the RGO, which is consistent with the observed upward shift in the 2D band position. The migration of the 2D band from 2705 cm^−1^ (single layer) to 2730 cm^−1^ (20 layers) further supports the multi-layered nature of the prepared RGO^48^.

**Fig. 5:**
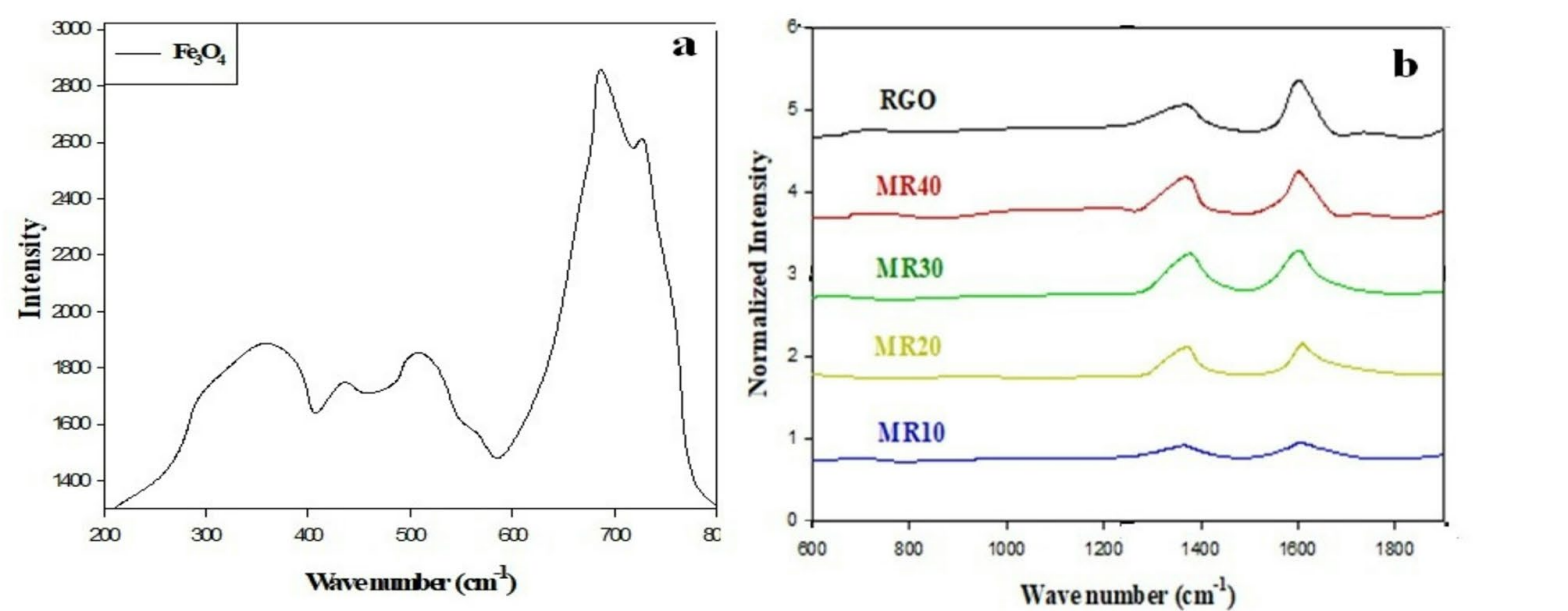
(**a**) The Raman spectrum for Fe_3_O_4_. (**b**) The Raman spectra for RGO, MR10, MR20, MR30 and MR40.

In addition to XRD measurements, Raman spectroscopy was performed to evaluate structural properties of Fe_3_O_4_ and Fe_3_O_4_@RGO hybrid nanocomposite samples. An excitation wavelength of 633 nm was utilized to prevent oxidation or phase transitions that could occur upon measurement due to the relatively high energy level. The Raman spectrum of pure Fe_3_O_4_ nanoparticles (see Fig. 5a) showed five unique bands corresponding to the T_2_g(1), Eg, T_2_g(2), T_2_g(3), and A_1_g vibrational modes of magnetite located at 195, 304, 462, 541, and 665 cm^−1^, respectively. In comparison, the Raman spectra of maghemite (γ-Fe_2_O_3_) are generally described as having three bands at 352, 501, and 703 cm^−1^; none of these bands were observed in the data obtained from this study, further supporting the conclusion that magnetite was present in the sample^49,50^. In addition to observing the presence of RGO using the D and G bands at 1363 cm^−1^ and 1592 cm^−1^, respectively, see Fig. 5b, the presence of magnetite was verified through the observation of an A_1_g band at 665 cm^−1^, which is indicative of the presence of magnetite on the surface of RGO^51^. The relative intensities of the D and G bands of RGO decreased progressively as the proportion of Fe_3_O_4_ increased in the composites. Additionally, the positions of the D and G bands shifted slightly to lower wavenumbers (blue-shifted). These position shifts can be attributed to the significant interaction occurring at the interface between the Fe_3_O_4_ nanoparticles and the RGO framework resulting in localized strain and altered electron–phonon coupling in the *sp*^*2*^ system^52^. These results collectively demonstrate the successful formation of RGO hybrids with attached magnetite particles, exhibiting strong structural correlation and proximity between the magnetic and conducting components, thus providing additional support for the enhanced electromagnetic performance of the composites.

#### Transmission electron microscopy

**Fig. 6:**
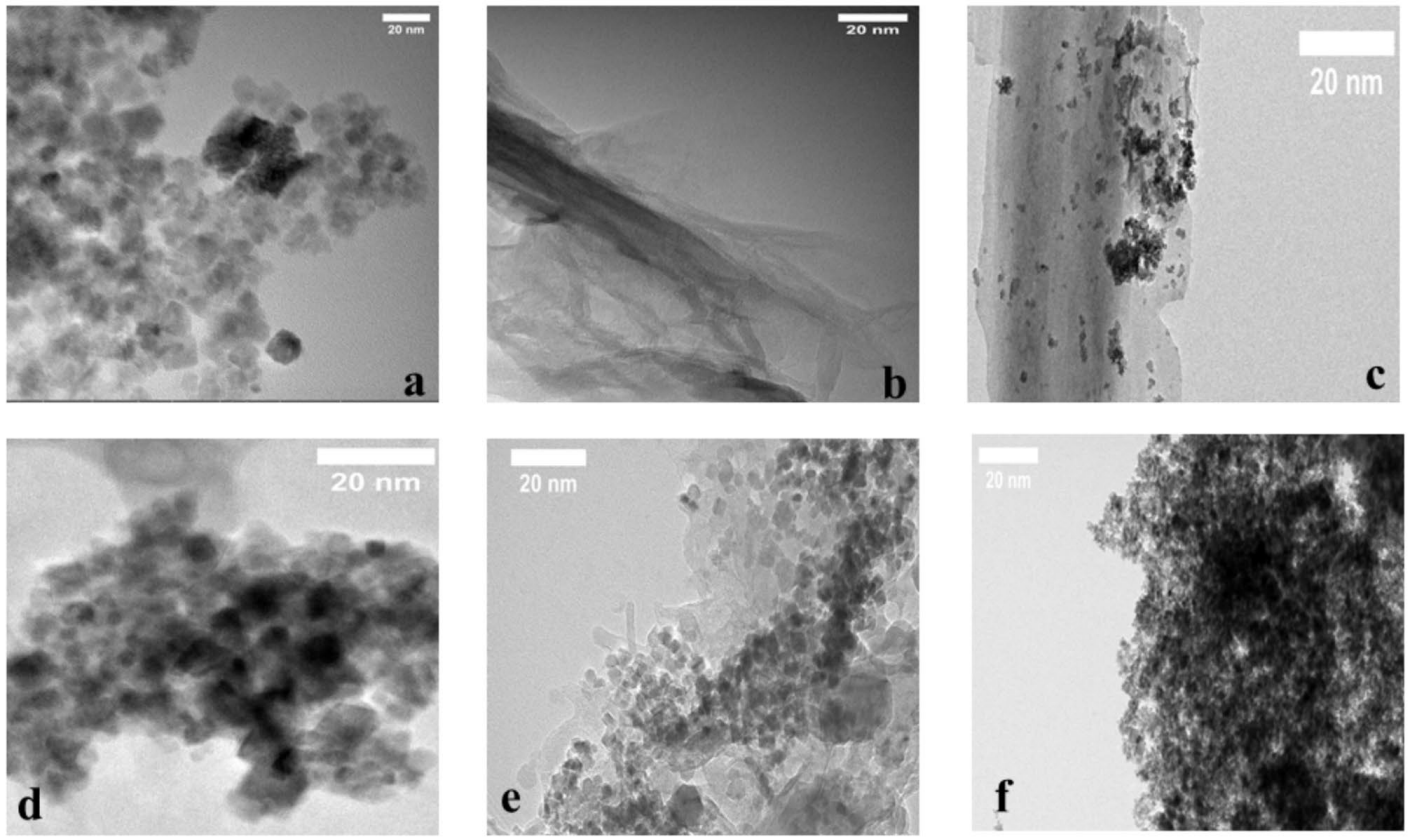
TEM micrographs of (**a**) Fe_3_O_4_ nanoparticle clusters, (**b**) RGO sheets, The Fe_3_O_4_@RGO hybrids’ TEM images were represented by panels (**c**–**f**) as MR10, MR20, MR30, and MR40, respectively.

The transmission electron micrographs shown in Fig. 6 present the morphology of Fe_3_O_4_ nanoparticles, RGO and the various Fe_3_O_4_@RGO hybrid nanocomposite architectures produced using different ratios of RGO to Fe_3_O_4_ (MR10, MR20, MR30 and MR40). In addition to illustrating the structural features of both the Fe_3_O_4_ and RGO components, TEM was used to provide morphological evidence for the formation of hybrid nanostructures through the interfacial association of the two materials. In particular, TEM images of the Fe_3_O_4_ component presented in Fig. 6a demonstrate that the particles are quasi-spherical in shape and have a narrow size distribution with a mean diameter of approximately 10 ± 5 nm; this is indicative of efficient control over nucleation and particle growth during the synthesis of the iron oxide via the co-precipitation method. The TEM image of RGO depicted in Fig. 6b provides further support for the crumpled and layered morphology commonly associated with reduced GO and demonstrates that it has been successfully exfoliated into individual sheets during the production of GO. The presence of uniformly distributed Fe_3_O_4_ nanoparticles attached to the surface of the RGO sheets (Fig. 6c–f) supports strong interfacial interactions between the magnetic material and the carbon-based electrical conductor and serves to act as attachment sites for other Fe_3_O_4_ nanoparticles, thereby inhibiting agglomeration of these entities and facilitating their uniform dispersion within the composite material structure^53^.

#### Vibrating sample magnetometer analysis

**Fig. 7:**
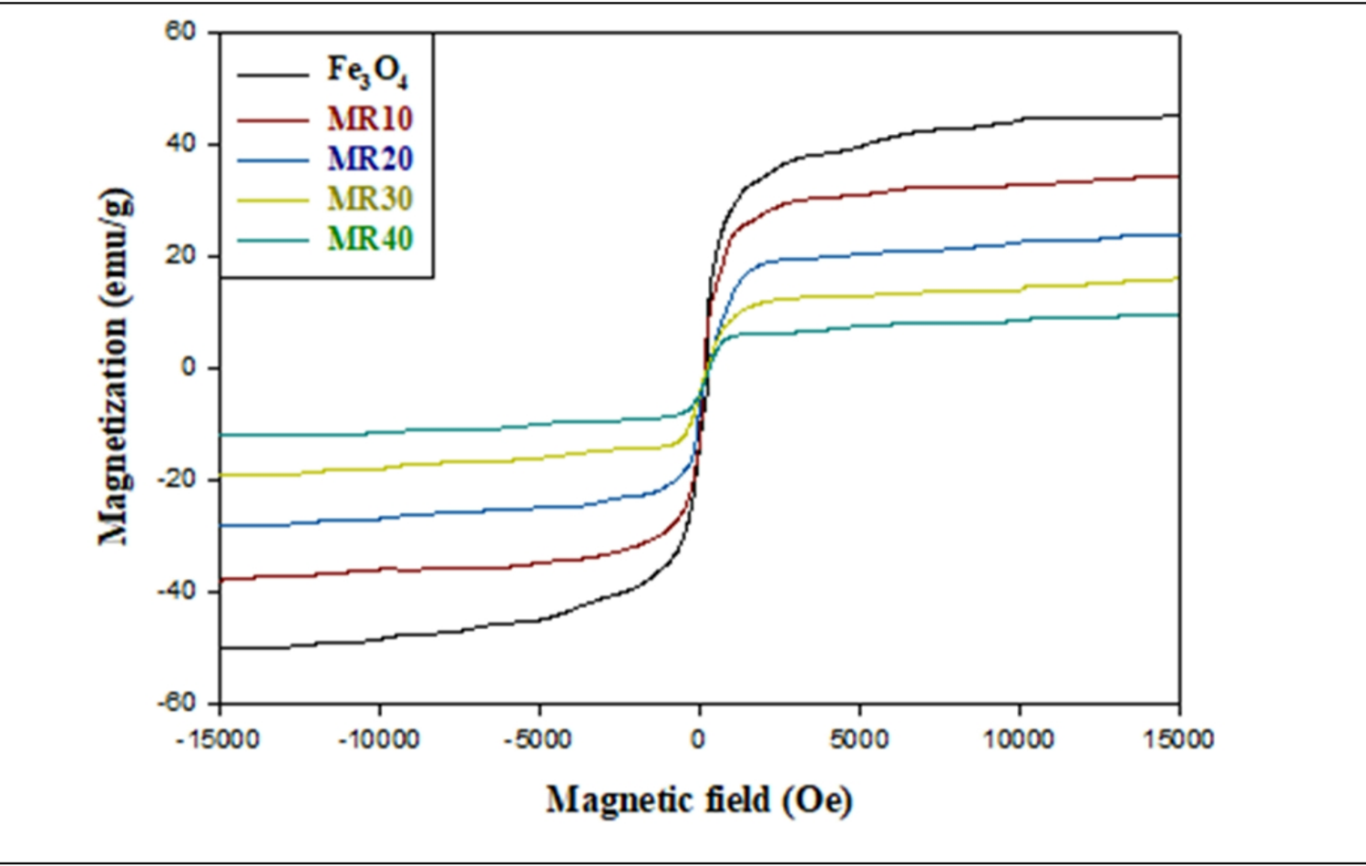
The hysteresis curves for Fe_3_O_4_, MR10, MR20, MR30, and MR40.

Vibrating Sample Magnetometry (VSM) was employed to examine the magnetic characteristics of Fe_3_O_4_ alongside the Fe_3_O_4_@RGO hybrid nanocomposites (MR10, MR20, MR30, and MR40) at a temperature of 298 K. Figure 7 displays the hysteresis loops obtained with each sample, while Table 2 shows the associated magnetic parameters of each sample; namely saturation magnetization (M_s_), remanent magnetization (M_r_), and coercivity (Hc) values. Hysteresis loops of all the samples recorded were narrow and symmetrical which are characteristics of ordinary soft magnetic materials^54^. The saturation magnetization of the pure Fe_3_O_4_ sample was the highest measured (M_s_ = 47.145 emu/g) with a gradual decrease in value noted with an increase in RGO content having values of 35.25, 24.11, 13.86 and 11.00 emu/g for MR10, MR20, MR30 and MR40 respectively. This systematic decrease in M_s_ values would be attributed to the diamagnetic effect of RGO which in effect decreases the total magnetic moment of the composite materials through a decrease in the relative amount of magnetic Fe_3_O_4_ per mass unit^55^. Similarly the remanent magnetization (M_r_) decreased from 2.15 emu/g in the pure Fe_3_O_4_ to values of 0.887, 0.658, 0.524 and 0.198 emu/g with MR10, MR20, MR30 and MR40 respectively. The decrease in M_s_ and M_r_ noted as the RGO fraction was increased in the composites indicates the strong coupling present at the magnetic–nonmagnetic interface between the Fe_3_O_4_ nanoparticles and the RGO sheets not allowing for unlimited spin orientation and weakening of the magnetic exchange interactions. All of the samples exhibited very low coercivity value (Hc < 100 Oe) indicating there is low magnetic hysteresis and the magnetization is reversible. Such behavior is characteristic of superparamagnetic materials, in which thermal effects at room temperature are sufficient to randomize the magnetic moments in the absence of an applied magnetic field^56^. The combination of soft magnetic behavior, high saturation magnetization, and negligible remanence makes the Fe_3_O_4_@RGO hybrids particularly suitable for applications that require rapid magnetic response and efficient electromagnetic absorption, such as EMI shielding and microwave attenuation.

**Table 2:** A summary of the magnetic properties of the Fe_3_O_4_ and Fe_3_O_4_@RGO composites.

Magnetic properties	Saturation magnetization (Ms, emu/g)	Magnetic remanence (Mr, emu/g)	Coercivity (Hc, Oe)
Fe_3_O_4_	47.154	2.147	20.332
MR10	35.248	0.887	27.741
MR20	24.113	0.658	40.009
MR30	13.857	0.524	61.358
MR40	11.001	0.198	77.249

### **Composite characterization**

#### FT/IR analysis

**Fig. 8:**
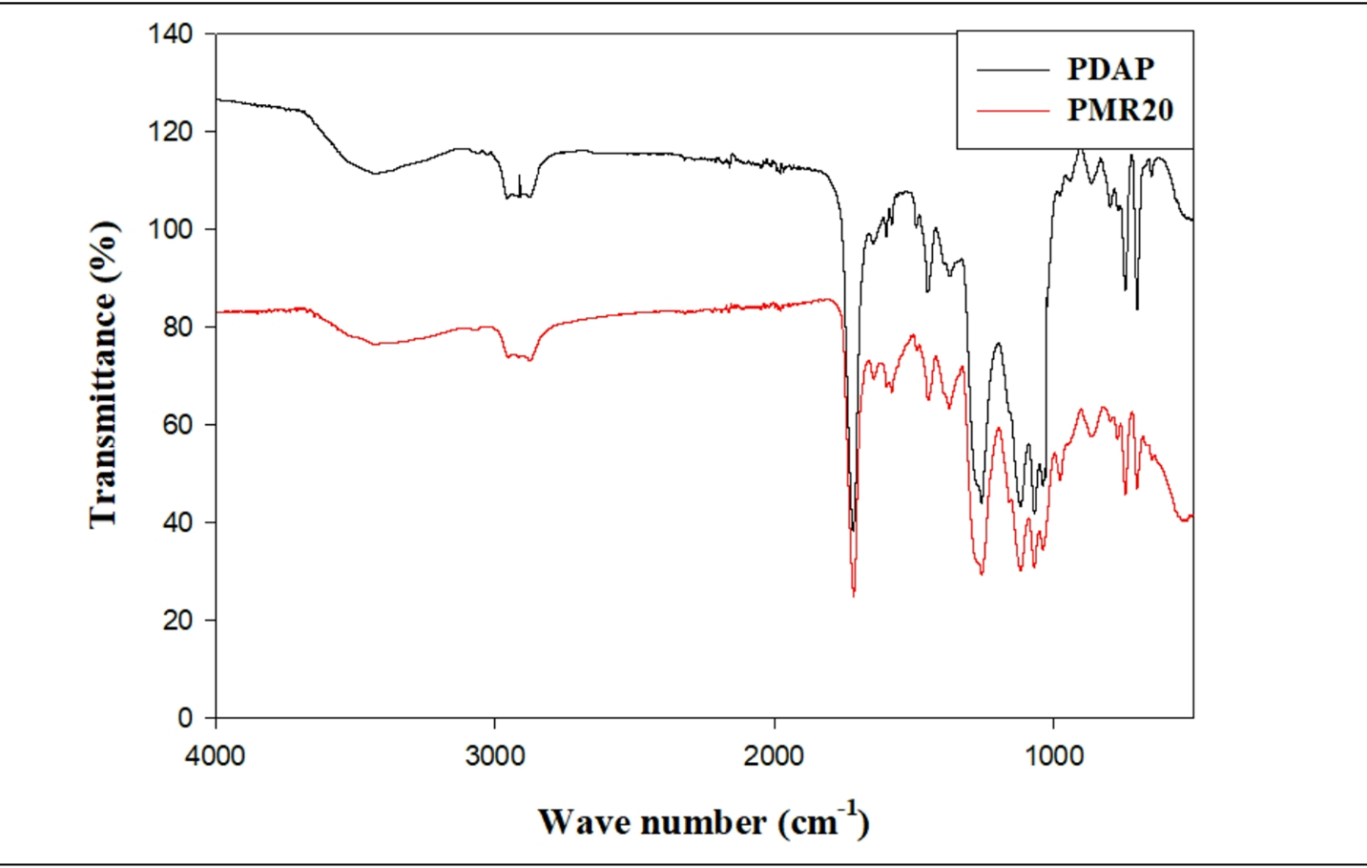
The FT/IR spectra for PDAP, Graphite, RGO and PMR20.

To examine the chemical composition of PDAP along with potential interactions involving Fe_3_O_4_@RGO fillers and the PDAP framework, Fourier-transform infrared (FT/IR) spectroscopy was utilized. Figure 8 presents the FT/IR spectra for unmodified PDAP as well as the exemplary composite PMR20. In the case of pure PDAP, key absorption signals emerged at 3427 cm^−1^, linked to O–H stretching vibrations; prominent bands at 3156 and 3123 cm^−1^ related to aromatic C–H stretching, whereas those at 2956 and 2923 cm^−1^ connected to aliphatic C–H stretching modes. A distinct, robust peak at 1717 cm^−1^ corresponded to C=O stretching within ester connections, and the signal at 1600 cm^−1^ aligned with aromatic C=C stretching vibrations. Signals from C–H deformation developed in the range 1451–1373 cm^−1^ along with C–O stretching from the ester units in the range 1259–1039 cm^−1^^57^. The FT/IR data for PMR20 paralleled the significant bands observed in pure PDAP, but with only slight shifts of the peak positions in some regions, near 924, 1690 and 3300 cm^−1^ being particularly affected. The absence of new bands and great reductions in the existing ones suggest that no chemical reactions have occurred between the polymer structure and the Fe_3_O_4_@RGO fillers. The changes in the position of the bands must therefore be due to some physical interactions and hydrogen bonding with oxygenated functional groups present on the surface of the Fe_3_O_4_@RGO and the ester groups or hydroxyl groups of the PDAP^58^. This constitutes evidence that the nanoparticles of Fe_3_O_4_@RGO become physically incorporated into the polymer matrix of PDAP with effective distribution owing to relatively weak interfacial interactions but not by covalent bonding. The similarity of the spectral properties of the PDAP indicated the stability of the structural characteristics of the backbone of the polymer, while the relatively small displacements of the band positions provided the evidence of interfacial polarization effects which are advantageous for EMI shielding purposes. Furthermore, the FT/IR data confirmed the presence of functional groups associated with the phthalate and allyl residues of the PDAP. Signals at 1725, 1275 and 1125 cm^−1^ corresponded to C=O and C–O–C stretching vibrations having their origin in the phthalate units, while the C=C stretching in the allylic group was around 1645 cm^−1^ which was accompanied by C=C vibrations in the aromatic rings at around 1598 cm^−1^. The observed decrease in intensity of 1645 cm^−1^ band during the polymerization process of di-allyl phthalate indicated the involvement of allyl groups and established the presence of cross-linked polymer systems. A slight shift in the C=O stretching band of PDAP and the appearance of broadened O–H features indicate hydrogen bonding and electrostatic interactions between PDAP chains and Fe_3_O_4_@RGO hybrid surfaces. This confirms the good interfacial compatibility between the matrix and the hybrid filler. Finally, the results of the FT/IR studies provided evidence for the successful formation of PDAP-derived nanocomposites which were characterized by significant physical interaction between the Fe_3_O_4_@RGO fillers and the polymer system which provided for uniform distribution and excellent interfacial interaction.

#### Scanning electron microscopy analysis

**Fig. 9:**
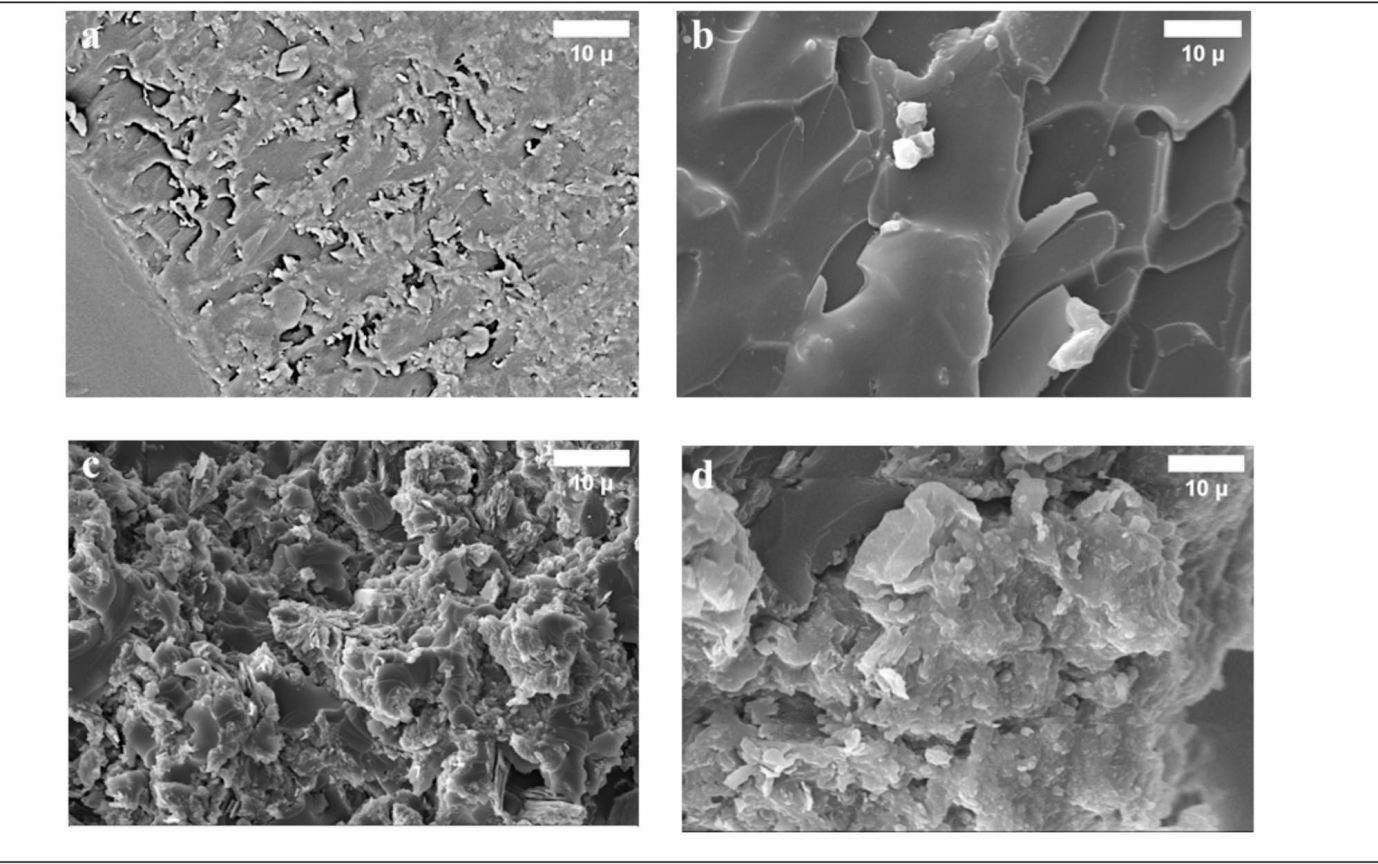
The SEM micrographs for **a**) PDAP (neat), **b**) P_1_MR20, **c**) P_2_MR20 and **d**) P_3_MR20.

The results from the characterization via scanning electron microscope (SEM) analysis of the pristine PDAP polymer and its consequent use in Fe_3_O_4_@RGO/PDAP nano-composites are given in Fig. 9. The surface of the pristine PDAP polymer (Fig. 9a) reveals a smooth and homogeneous surface structure with no evident disconnected particle structures which are indicative of the homogeneous nature of the pristine bulk polymer matrix. The presence of Fe_3_O_4_@RGO fillers (Fig. 9b–d) gives a decidedly different picture to the surface morphology of the materials. The Fe_3_O_4_ nano-particles appear to be anchored upon the wrinkled RGO nano-sheets and appear to be well distributed throughout the PDAP matrix giving rise to connected conductive–magnetic interpenetrating streaks in the polymeric bulk. The effect of this component of intermixing is that the structures of microstructure of the polymer is rendered more complex and an efficient interface for the formation of charge and dipole polarization is enhanced. At the higher loadings of fillers some partial aggregation of the nano-particles and localized clustering occurs particularly in the case of the higher concentrations of Fe_3_O_4_/RGO combinations. These morphological holds are in correlation with the lowered-crystallinity of the X-ray results and are borne out by the knowledge that ample amounts of additions of fillers limits the primal capability of the matrix to evenly hold the nano-particles. The Fe_3_O_4_ nano-particles are also still well distributed upon the surfaces of the RGO to the extent resulting in avoidance of forms of too large agglomeration and affording benefit of strong interfacial adhesion between the polymeric chains and the hybrid fillers. This dual contribution uniform dispersion of conductive RGO sheets and magnetic Fe_3_O_4_ nanoparticles facilitates the formation of continuous conductive pathways and magnetic loss centers, thereby improving both reflection and absorption mechanisms in EMI shielding. Among all compositions, the composite containing 20 wt% RGO (PMR20) displays the most homogeneous morphology with minimal aggregation, correlating directly with its superior shielding effectiveness compared to other loading ratios.

#### Electrical conductivity and EMI shielding effectiveness

**Fig. 10:**
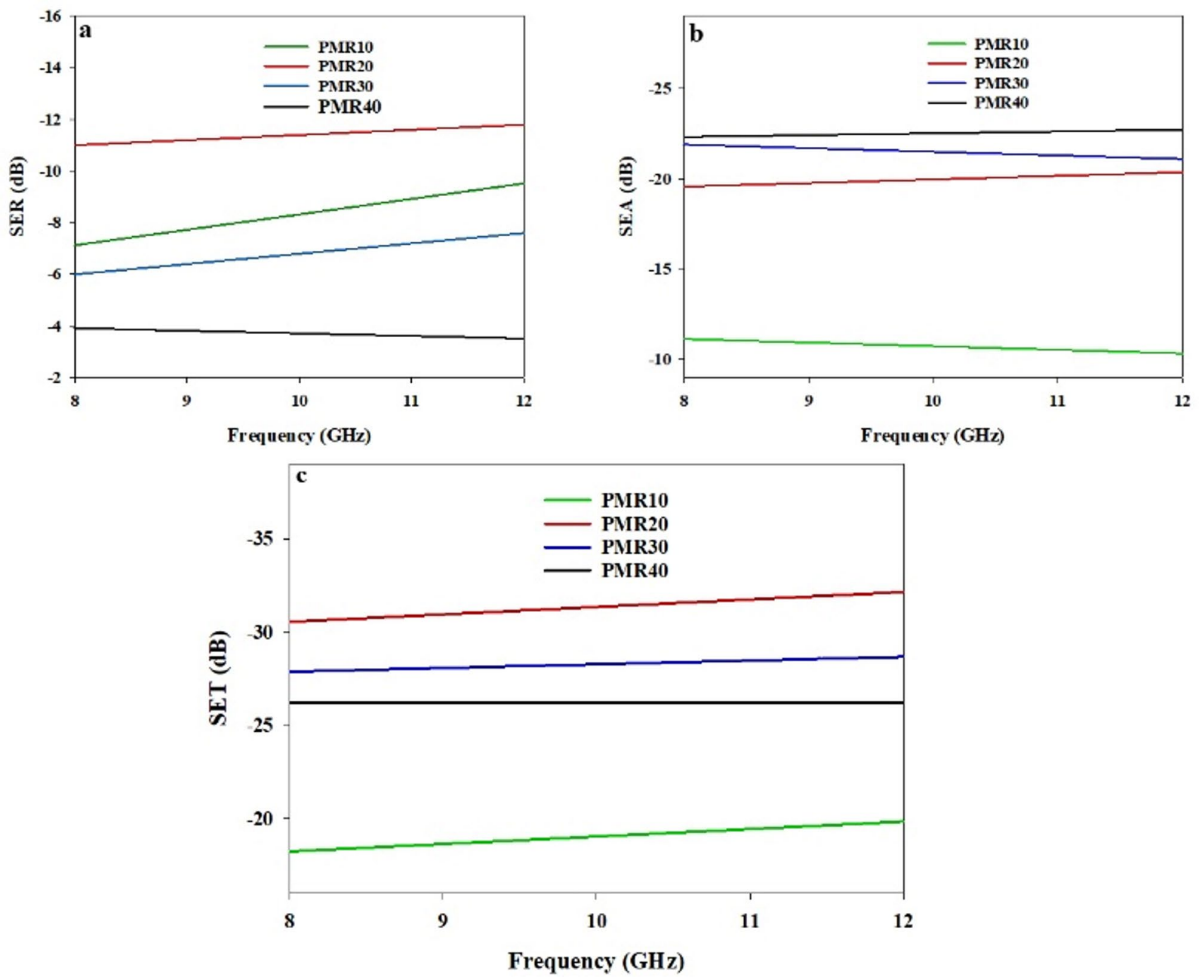
(**a**) SER, (**b**) SEA and (**c**) SET as a function of the frequency for PMR10, PMR20, PMR30, and PMR40.

**Fig. 11:**
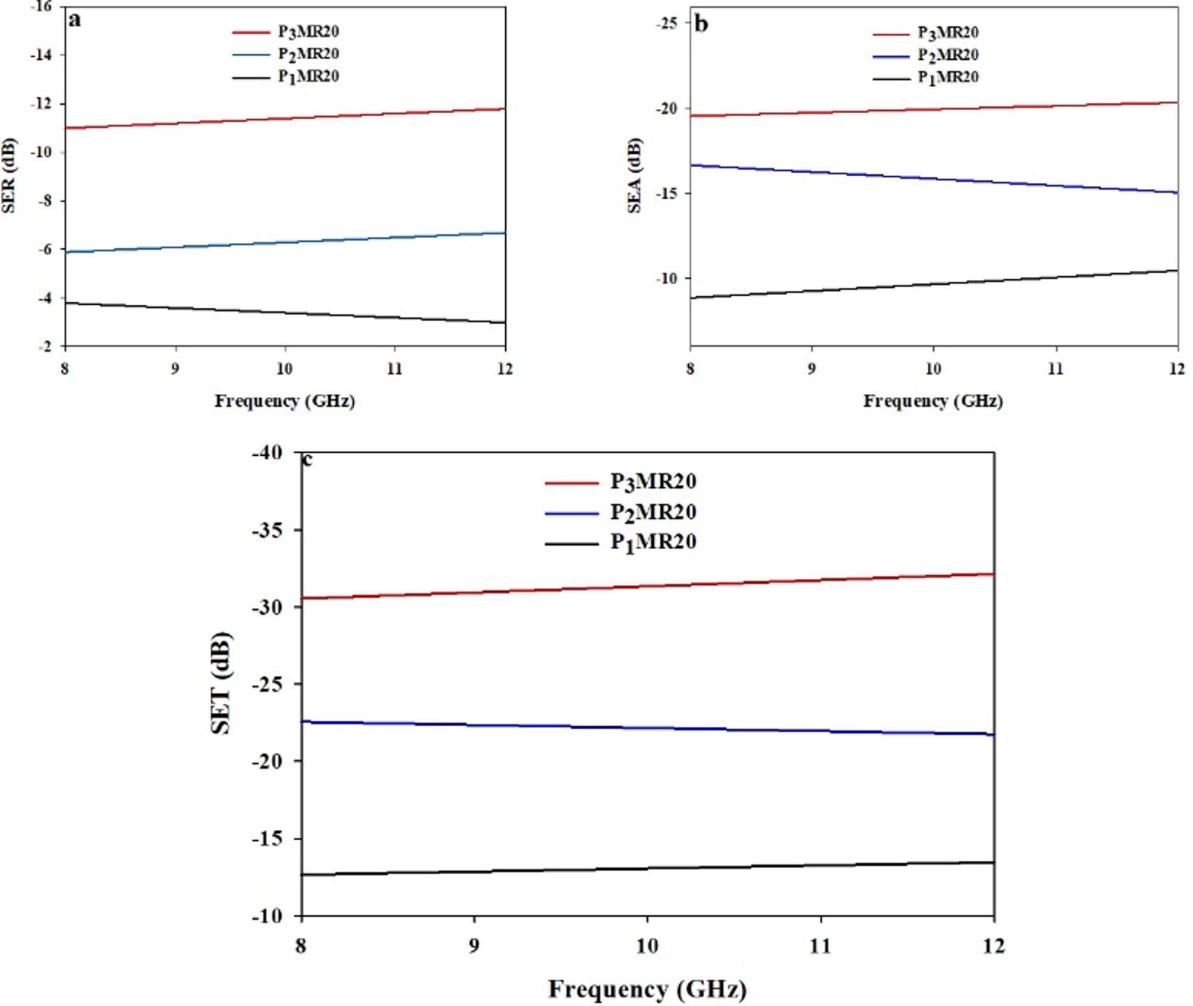
(**a**) SER, (**b**) SEA, (**c**) SET as a function of the frequency for P_1_MR20, P_2_MR20, and P_3_MR20.

**Fig. 12:**
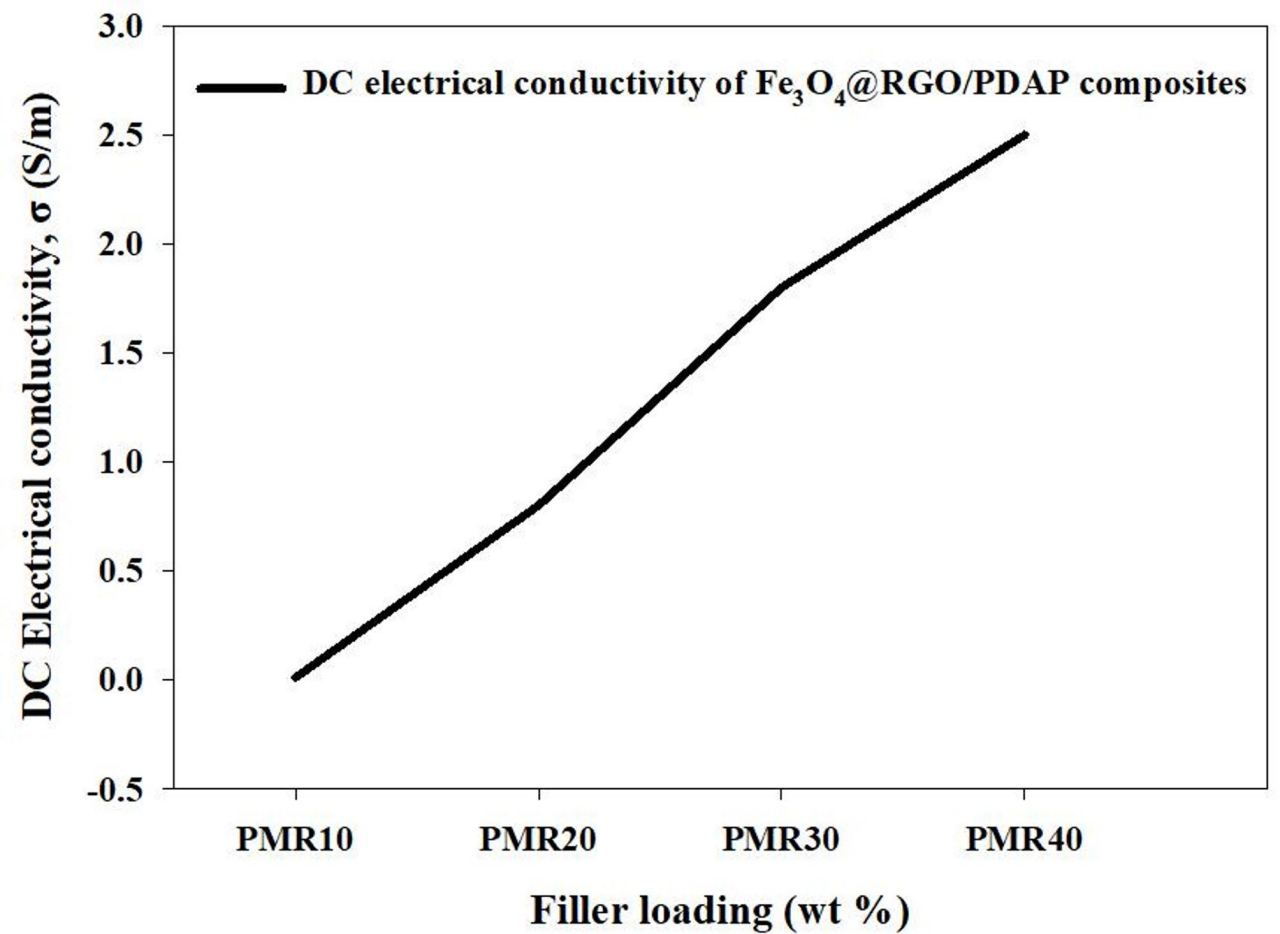
Variation of DC electrical conductivity (σ) of Fe_3_O_4_@RGO/PDAP composites with hybrid filler loading.

The EMI shielding effectiveness (SE) of the Fe_3_O_4_@RGO/PDAP nanocomposite depends upon both the composition of the Fe_3_O_4_@RGO hybrid filler and the loading level of the hybrid filler within the PDAP matrix. To identify the appropriate composition, two separate sets of experiments were performed. In the first set, the amount of hybrid filler within the PDAP was held constant as the proportion of RGO to Fe_3_O_4_ within the hybrid filler varied. In the second set, the RGO: Fe_3_O_4_ composition that resulted in the highest SE was then used to maximize the SE by optimizing the amount of hybrid filler within the PDAP. SER of each sample vs. the frequency of the radiation is depicted in Fig. 10A. Both a dependence on the frequency and the composition of the hybrid filler are evident. The average SER values for PMR10 and PMR20 were 9.9 dB and 11.4 dB, respectively. The average SER values for PMR30 and PMR40 were 6.7 dB and 3.6 dB, respectively. These trends in SER can be attributed to changes in the shape of the hybrid filler particles and the interactions between the Fe_3_O_4_ particles and the RGO sheets and the radiation. As illustrated in Fig. 10B, the SEA as a function of the frequency of the radiation increases with increasing RGO content and is clearly dependent on the composition of the hybrid filler. The average SEA values for PMR10 and PMR20 were 10.7 dB and 20.1 dB, respectively. The average SEA values for PMR30 and PMR40 were 21.3 dB and 22.5 dB, respectively. The increase in SEA with RGO content is due to the improvement in electrical conductivity of the Fe_3_O_4_@RGO composites which enhances the polarization of the electric field and the losses at the interfaces within the PDAP. The total shielding effectiveness (SET) as a function of the frequency of the radiation from 8 to 12 GHz is shown in Fig. 10C. The average SET values for PMR10, PMR20, PMR30 and PMR40 were 18.9 dB, 31.5 dB, 28.4 dB and 26.3 dB, respectively. The highest value of SE was obtained for PMR20 with an average SET of about 31.5 dB. This is likely due to the synergy of the effects of the Fe_3_O_4_ (which results in a magnetic loss mechanism and contributes to the reflection of the incident EM waves) and RGO (which contributes to the absorption of the incident EM waves through its high electrical conductivity). As the RGO content is further increased, the excess RGO sheets may result in reduced effective paths for the magnetic fields leading to a decreased SE^59^. To further assess the influence of total filler loading, Fe_3_O_4_@RGO with the optimized composition (MR20) was incorporated into PDAP at different weight percentages. The corresponding SE parameters are shown in Fig. 11. SER values increased steadily with increasing MR20 loading, averaging 3.4 dB, 6.3 dB, and 11.4 dB for PDAP composites containing 10 wt%, 20 wt%, and 25 wt% MR20, respectively. SEA followed the same trend, with average values of 9.7 dB, 15.9 dB, and 20.1 dB across the X-band. The resulting SET (Fig. 11c) increased from 13.2 dB to 22.3 dB and 31.5 dB, respectively, confirming the dominant contribution of absorption in high-filler composites^60^. This improvement can be explained by the increased proximity of RGO sheets and magnetic Fe_3_O_4_ domains within the PDAP matrix, which promotes the formation of interconnected conductive–magnetic networks. These networks facilitate multiple internal reflections, interfacial polarization, and magnetic dipole resonance, thereby enhancing both absorption and reflection components of EMI attenuation^61^. Although a full NRW retrieval was not experimentally feasible with the available setup, the analytical framework of the Nicolson–Ross–Weir (NRW) method was adopted conceptually to calculate the real and imaginary parts of permittivity and permeability. Using the measured SET, SEA, and SER values, together with the standard attenuation constant and impedance relations, ε′, ε″, µ′, and µ″ were calculated and used to interpret the dielectric and magnetic loss contributions. This approach provides a physically consistent estimation of the electromagnetic parameters analogous to NRW theory. To quantitatively interpret the dominant absorption contribution in the Fe_3_O_4_@RGO/PDAP composites, the key electromagnetic parameters were calculated directly using classical electromagnetic relations at 10 GHz. The calculated permittivity values (ε′ = 12.0, ε″ = 4.0) indicate strong dielectric polarization and conduction loss associated with the partially percolated RGO network and the Maxwell–Wagner interfacial polarization at PDAP/RGO and PDAP/Fe_3_O_4_ interfaces. The calculated permeability values (µ′ = 1.30, µ″ = 0.30) reflect magnetic relaxation processes, including natural resonance and multi-domain wall damping in the Fe_3_O_4_ nanoparticles. The dielectric and magnetic loss tangents, calculated as tanδe = ε″ / ε′ ≈ 0.33 and tanδm = µ″ / µ′ ≈ 0.23, confirm the coexistence of strong dielectric and magnetic loss channels. From these parameters, the attenuation constant was calculated as α ≈ 225 Np/m, while the corresponding skin depth was found to be δ ≈ 3 mm. Since the composite specimens used in this study have a thickness of approximately 5 mm (> δ), the majority of the incident electromagnetic energy penetrates the surface and is dissipated inside the material. This directly explains the experimentally observed absorption-dominated behavior (SEA ≫ SER). The Fe_3_O_4_@RGO hybrid filler plays a synergistic role: (i) RGO forms conductive pathways that enhance conduction loss, interfacial polarization, and dipole relaxation; (ii) Fe_3_O_4_ introduces magnetic resonance and eddy current damping; (iii) the combined dielectric–magnetic effects improve impedance matching (Z ≈ Z_o_), allowing more energy to enter the material rather than being reflected. These combined mechanisms account for the high attenuation efficiency and the superior absorption-dominated EMI shielding performance of the PDAP-based hybrid nanocomposites. The DC electrical conductivity (σ) of the Fe_3_O_4_@RGO/PDAP composites shows a strong dependence on the filler loading^62^, as presented in Fig. 12. A slow conductivity response is observed for the PMR10 sample (σ ≈ 1 × 10^−2^ S/m), indicating that the conductive network formed by the hybrid Fe_3_O_4_@RGO filler has not yet become continuous. Upon increasing the loading to 20 wt% (PMR20), the conductivity increases abruptly to ~ 0.8 S/m, representing nearly two orders of magnitude enhancement. This sharp transition is characteristic of a percolation-like behavior^63^, where the conductive pathways of RGO sheets begin to interconnect and facilitate long-range electron hopping^64^. Further increasing the hybrid filler content leads to a more progressive rise in σ, reaching ~ 1.8 S/m and ~ 2.5 S/m for PMR30 and PMR40, respectively. These trends clearly indicate that the electrical percolation threshold (ϕc) occurs slightly above 10 wt% and close to 15 wt% Fe_3_O_4_@RGO, where a fully connected conductive network is established within the PDAP matrix. The observed DC conductivity evolution correlates strongly with the imaginary permittivity (ε″) and with the absorption component of EMI shielding (SEA). A higher σ enhances conduction loss and Maxwell–Wagner interfacial polarization, promoting stronger dielectric loss, while the Fe_3_O_4_ nanoparticles contribute magnetic relaxation^65^. The synergy between these effects enables efficient attenuation of the incident electromagnetic waves, as confirmed by the calculated dielectric and magnetic loss tangents and the attenuation constant. These results demonstrate that the formation of a percolated RGO-based network at filler loadings ≥ 20 wt% plays a dominant role in governing the absorption-driven EMI shielding performance of the Fe_3_O_4_@RGO/PDAP nanocomposites. The uniform microstructure observed in the SEM images of PMR20 further supports these findings, confirming the effective dispersion of hybrid fillers. A comparative summary of the EMI shielding performance of this system with previously reported Fe_3_O_4_ and graphene-based polymer composites is provided in Table 3, highlighting the superior performance of the Fe_3_O_4_@RGO/PDAP system at relatively low filler loadings.

**Table 3:** Comparison of the electromagnetic interference shielding effectiveness (SE) of Fe_3_O_4_@RGO/PDAP composites with previously reported polymer-based systems in the X-band frequency range (8–12 GHz).

Matrix	Filler	Filler Loading (%)	Thickness (mm)	Freq.Range (GHz)	SE(dB)	References
PVA	Fe_3_O_4_@RGO	35 wt %	3	8–12	− 15	^66^
PVC		10 wt%	1.8		− 13	^67^
PS		2.24 v%	–		− 30	^68^
PU		25 wt%	6		− 23.2	^2^
PDAP		5 wt%	5		− 13.2	This work
PDAP		15 wt%	5		− 22.3	
PDAP		25 wt%	5		− 31.5	
PP	MWCNT	30 wt%	–		− 40.3	^69^
PU	Ni, Co@MWCNT	25 wt%	–	18-26.5	− 35	^70^
EOC	MWCNT	10 wt%	2	8–12	− 25.5	^71^
Epoxy	RGO-SWCNT	15 wt%	–	1–18	− 50	^72^
PMMA-PVDF	RGO	4 wt%	–	8–12	− 33.3	^73^
Carbon fiber fabrics	Ag-RGO	5 wt%	–	8–12	− 22.68	^74^

As shown in Table 3, the Fe_3_O_4_@RGO/PDAP composites exhibit competitive shielding effectiveness compared to recently reported graphene-based systems, while offering absorption-dominated behavior and a thermosetting PDAP matrix that has rarely been explored for EMI shielding.

## Conclusions

In summary, Fe_3_O_4_@RGO hybrid nano-fillers were successfully synthesized and uniformly integrated into a poly(di-allyl phthalate) (PDAP) matrix to produce lightweight EMI shielding composites with well-controlled dielectric–magnetic coupling. Structural analyses confirmed the effective anchoring of Fe_3_O_4_ nano-particles onto RGO sheets and their homogeneous distribution within the polymer. The calculated electromagnetic parameters (ε′ ≈ 12, ε″ ≈ 4, µ′ ≈ 1.3, µ″ ≈ 0.3 at 10 GHz) revealed strong dielectric and magnetic dissipation channels, which, together with the enhanced interfacial polarization and conduction pathways provided by RGO, resulted in an absorption-dominated shielding mechanism. This dominance of absorption is consistent with the high attenuation constant and the condition SEA ≫ SER across the X-band. The PMR20 composition exhibited the highest average SET (~ 31.5 dB), corresponding to over 99.9% attenuation of incident radiation. Overall, the Fe_3_O_4_@RGO/PDAP system demonstrates efficient dielectric–magnetic synergy, good impedance matching, and favorable absorption characteristics, establishing PDAP-based hybrids as promising candidates for lightweight EMI shielding. Future work will focus on mechanical evaluation and long-term stability to further support their practical implementation.

## Data Availability

The data and materials of this study are declared to be available by the authors. Interested parties can request for the datasets generated during the study from the corresponding author. (Hussein Oraby [hussein.mohamed4544@yahoo.com] (mailto: hussein.mohamed4544@yahoo.com) ) on reasonable.
